# Curcumin modulates cellular AP-1, NF-kB, and HPV16 E6 proteins in oral cancer

**DOI:** 10.3332/ecancer.2015.525

**Published:** 2015-04-23

**Authors:** Alok Mishra, Rakesh Kumar, Abhishek Tyagi, Indu Kohaar, Suresh Hedau, Alok C Bharti, Subhodeep Sarker, Dipankar Dey, Daman Saluja, Bhudev Das

**Affiliations:** 1 Institute of Cytology and Preventive Oncology (ICMR), I–7, Sector-39, Noida 201301, India; 2 Ambedkar Centre for Biomedical Research (ACBR), University of Delhi, Delhi 110 007, India; 3 National Cancer Institute, NIH, Bethesda, MD, USA 20892; 4 Mayo Clinic, Rochester, MN, USA 55905; 5 Amity University, Noida 201303, India; * Rakesh Kumar deceased.

**Keywords:** activator protein-1 (AP-1), curcumin, human papilloma virus (HPA), nuclear factor kappaB (NF-kB), transcription

## Abstract

In this study, we investigated the effects of the natural antioxidant curcumin on the HPV16-positive oral carcinoma cell line 93VU147T and demonstrated that curcumin is not only a potent inhibitor for the activity of host nuclear transcription factors AP-1 and NF-kB but it also selectively suppresses transcription of the HPV16/E6 oncogene during the carcinogenic process in oral cancer cells. This study suggests a therapeutic potential of curcumin for high-risk human papilloma virus (HPV)-infected oral cancers.

## Introduction

Oral squamous cell carcinoma (OSCC) is the sixth most common cancer and accounts for approximately 5% of all malignant tumours worldwide. In India and South East Asia, it is the most common malignancy accounting to 50% of all malignant tumours [1]. Most OSCC is attributed to smoking and alcohol consumption, whereas a proportion of oral cancers have been demonstrated to contain anogenital human papillomavirus (HPV) infections. Many studies have established that the high-risk HPVs, especially HPV type 16 (HPV16), are aetiologically related to a subset of head and neck squamous cancer cell [2, 3].

*Cis* regulatory elements located at the upstream regulatory region (URR) of the HPV genome is bound by a large number of host-cell transcriptional factors and their different combinatorial complexes play a key role in the transcriptional regulation of downstream E6 and E7 oncogenes’ expression. The interplay of viral- and host-encoded transcription factors orchestrate the ultimate regulation of transcription machinery of the HPVs [4, 5]. Nuclear transcription factors activator protein-1 (AP-1) and nuclear factor kappa B (NF-kB) are implicated for efficient transcription of HPV16 [6–9] and also considered central host factors for tissue-specific transcription of HPVs [5, 9]. E6 and E7 oncoproteins are encoded by the high-risk HPVs, which bind and degrade the host tumour suppressor proteins p53 and retinoblastoma (pRb), respectively, to facilitate HPV-induced carcinogenesis [10, 11].

Transactivation and the DNA-binding affinity of many transcription factors, such as NF-kB and AP-1, can be modulated by alterations of the intracellular redox status. Antioxidant-induced modifications of these transcription factors are obviously sufficient to interfere with the architecture of an HPV-specific transcription complex, resulting in a selective suppression of viral oncogene expression [12–14].

Curcumin (diferulolylmethane) has also been also shown to suppress the expression of many viral genes of human immunodeficiency virus (HIV) and HPV [15, 16]. It is an active component of perennial herb turmeric exhibits antitumour activity via multiple pathways of NF-kB and AP-1 [17–19, 23]. Retardation of tumorigenesis and reduction in DNA adducts by curcumin in oral cancer cells and leukoplakias have been reported previously [20, 21, 35, 36], but the effect of curcumin on HPV-harbouring OSCC is still unknown.

In our previous studies, we demonstrated that the transactivation and DNA-binding affinity of AP-1 and NF-kB can be modulated by alterations of the intracellular redox status by synthetic antioxidative agent PDTC [9] and natural antioxidant curcumin [16], which leads to selective suppression of transcription of HPV in cervical cancer cells. Therefore, we are tempted to investigate the role of curcumin in HPV16-positive oral cancer cell line-93VU147. This natural antioxidant-induced abolition of the HPV oncogene expression is mediated by the downregulation as well as the decreased transactivation of the AP-1 and NF-kB superfamily members and represents a novel mechanism that can regulate HPV-induced oral carcinogenesis.

## Materials and Methods

### Curcumin, cell line, and culture conditions

The human oropharyngeal squamous cell cancer cell lines 93VU147T was a kind gift from Dr Renske Steenbergen (VU Medical Center, Molecular Pathology Unit, Amsterdam, the Netherlands). Cells stably express the HPV16 E6 mRNA transcript and contain 1–2 integrated copies of HPV16 DNA per cell genome [24]. These cells were maintained in Dulbecco’s modified Eagle medium (DMEM) containing 10% foetal bovine serum (GIBCO Life Technologies Inc, Gaithersburg, MD) in a humidified (37°C, 5% CO2) incubator and passaged when they reached 80% confluence.

Curcumin was obtained from Sigma Chemicals (St. Louis, MO, cat no. C1386) and was freshly dissolved in ethanol and diluted in the medium immediately before use. Curcumin derived from *Curcuma longa* (Turmeric) powder and has many synonyms. Chemically, it is (E, E)-1,7-bis (4-hydroxy-3-methoxyphenyl)-1,6-heptadiene-3,5-dione, diferuloylmethane, diferulylmethane, natural yellow 3.

## 3-(4,5-Dimethylthiazol-2-yl)-2,5-diphenyltetrazolium bromide (MTT) assay

The cytotoxic effect of curcumin on cell line was determined by MTT dye uptake method. The cells were incubated in triplicate in a 96-well plate in the presence or absence of indicated test samples in a final volume of 0.1 ml for 24 h, 48 h, and 72h at 37°C. Thereafter, 0.025 ml of MTT solution (5 mg/ml in PBS) was added to each well, incubated at 37°C, the lysis buffer (20% SDS 50% dimethyl formamide) was added, and the extract was incubated overnight at 37°C for the solublisation of formazan crystals. The optical density (OD) at 570 nm was measured using a 96-well multiscanner autoreader (Biotek) with the lysis buffer serving as blank. The percentage of cell viability was calculated using the following formula: Percentage cell viability = (OD of the experiment samples/OD of the control) multiplied by 100.

### HPV detection by polymerase chain reaction

To test the positivity of HPV16 genome in 93VU147T cells, total genomic DNA was amplified using specific primers for HPV types 16 after DNA extraction by standard phenol/chloroform method as described earlier [25].

### Preparation of protein extract

Protein extracts from cells were prepared by the method of Dignam *et al* with certain modifications [26]. Briefly, the method involved washing of cells with cold 1X-PBS and suspending the pellet in ice-cold buffer A {20 mM HEPES pH = 7.6, 20% (v/v) glycerol, 10 mM NaCl, 1.5 mM MgCl_2_, 0.2 mM EDTA, 1 mM DTT, 1 mM PMSF, 2 mg/ml leupeptin, and 10 mg/ml aprotinin. The lysates were microfuged at 4000 rpm for 10 min at 4°C after incubation for 15 minutes on ice. The pellet containing isolated nuclei was resuspended in buffer B {20 mM HEPES pH 7.6, 25% (v/v) glycerol, 500 mM NaCl, 1.5 mM MgCl_2_, 0.2 mM EDTA, 1 mM DTT, 1 mM PMSF, 2 mg/ml leupeptin, and 10 mg/ml aprotinin} and microfuged after 1 h at 14,000 rpm at 4°C for 25 min. to obtain supernatant designated as nuclear extract.

### Electrophoretic mobility shift assay

Consensus oligonucleotides of AP-1: 5′CGCTTGATGACTCAGCCGGAA-3′, Oct-1: 5′-TGTCGAATGCAAATCACTAGAA-3′, and NF-kB: 5′AGTTGAGGGGACTTTCCC AGGCC-3′ were synthesised by Applied Biosystems and annealed oligonucleotides were labeled with [γ-^32^P] ATP (3000 Ci/mmol, Jonaki, India) with T4 polynucleotide kinase. The binding reaction and competition assays were performed to determine the specificity of DNA probes as described earlier [9]. For monitoring composition of AP-1, NF-kB, and Oct-1, following antibodies of Santa Cruz Biotechnology were used: c-Jun (sc-605), JunB (sc-73), JunD (sc-74), c-Fos (sc-253), FosB (sc-48), Fra-1 (sc-605), Fra-2 (sc-171), p50 (sc-114), p65 (sc-109), p52 (sc-298), c-Rel (sc-70), RelB (sc-226), Bcl-3 (sc-185) [25, 26].

### Immunoblotting

Protein extracts (25–50 μg/lane) were separated in 8–12% polyacrylamide gel and electrotransferred on poly(vinylidene fluoride) (PVDF) membranes (Millipore Corp, Bedford, MA, USA). The membrane was blocked with 5% milk and incubated overnight with primary antibody {(c-Jun (sc-605), JunB (sc-73), JunD (sc-74), p50 (sc-114), p65 (sc-109, p53 (sc- 126), E6 (sc-460), Bcl-2 (sc-7382), bax-2 (sc-526), and c-IAP2 (sc-1957)} at 4°C. These blots were washed, incubated with HRP- anti-rabbit IgG secondary antibodies and visualised by Luminol detection kit (Santa Cruz Biotech, USA). β-Actin expression was used for loading control.

### Hybridisation probes and northern blot hybridisation

Plasmid harbouring HPV16 gene was kindly provided by Peter Angel (DKFZ, Germany) and for β-actin by L. Kedes (Medical Center, Palo Alto, CA). HPV16/E6-specific probes were generated by polymerase chain reaction (PCR) for HPV16 E6 (nucleotide [nt] 83 to 559 with an amplicon size of 476 bp, (5′-GAA ACC GGT TAG TAT AAA AGC AGA C-3′ and 5′-AGC TGG GTT TCT CTA CGT GTT CT-3′) and random-labelled according to manufacturer’s protocol (Bangalore Genie, India). Total RNA was extracted by TRI reagent as per instruction manual (Sigma Inc, USA). Northern blotting was carried out by resolving approximately 15–20 ug of RNA on 1% agarose–MOPS–formaldehyde gel. Capillary blotted membrane was washed in 6X SSC, air-dried, exposed in phosphorimager (Fujifilm FLA-5100) after pre-hybridisation and hybridisation in Perfect HYB-PLUS (Sigma Inc, USA) solution as suggested by the manufacturer’s protocol.

## Results

**Figure 1.**
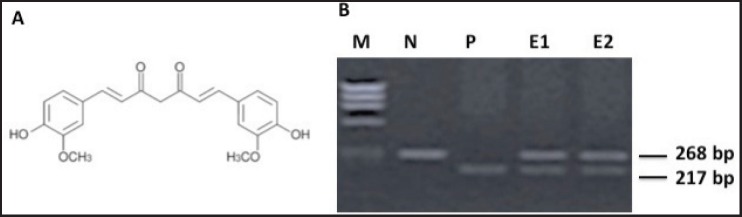
A) Chemical structure of antioxidant curcumin (diferuloylmethane) mentioned in materials and methods. B) PCR reaction confirming presence of HPV16 DNA in cultured oral cell line.

Figure 1A shows the chemical structure of the curcumin used for experiments and its reconstitution method is discussed previously. Prior to performing the experiments, the cell line was tested for the HPV16 genome by standard PCR (Figure 1B). A type-specific HPV-16 primer was used to detect 217 bp viral amplicon (Figure 1B, lane P), while the beta-globin primer (amplicon size 268 bp, lane N Figure 1B) was used as control to check DNA integrity. Lanes E1 and E2 were having total DNA from cells to detect HPV16 genome.

## Curcumin decreases cell viability and induces morphological changes in HPV16 positive oral cancer cell line 93VU147T

**Figure 2.**
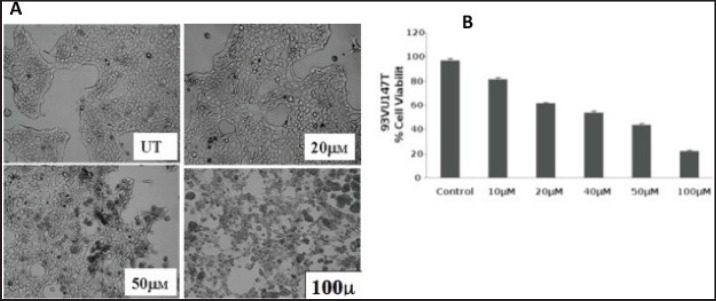
A) Curcumin changes morphology of HPV16 harbouring 93VU-147T oral cancer cells *in vitro* after treatment UT = Untreated. B) MTT assay showing % viability of cancer cells in post-treated condition of curcumin. uM = micromolar.

In the pilot experiments performed in triplicates, HPV 16-positive oral carcinoma cells, 93VU-147T was incubated in different concentrations of curcumin (0–100 uM) for a fixed period of 1 h AND for different time periods (15 min to 24 hr). After analysing the morphological features (Figure 2A), an optimum time of 3 h and a maximum nontoxic concentration of 100 uM curcumin were standardised for further experiments. The cell viability after the curcumin treatment was further verified by the standard MTT assay, showing > 85% cell death at 100 uM (Figure 2B). These data together showed the distinct decrease in cell viability of HPV16-infected oral cells in dose dependent manner by curcumin.

### Curcumin induces apoptosis in HPV-positive cells

**Figure 3.**
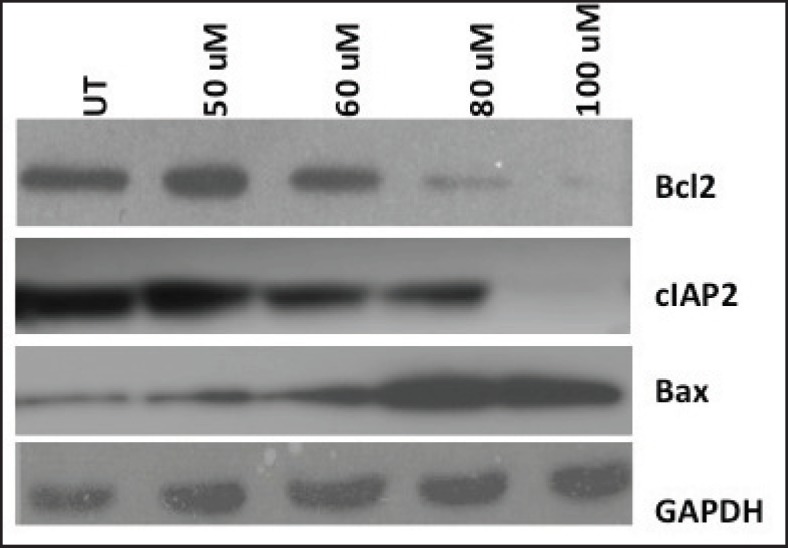
Induction of apoptosis of HPV harbouring oral cancer cells by antioxidant curcumin. Anti-apoptotic proteins Bcl-2 and cIAP-2 decreased, while pro-apoptotic marker Bax increased gradually with the increasing concentration of curcumin dosage. Glyceraldehyde 3-phosphate dehydrogenase (GAPDH) level, used as loading control was unaffected post-treatment and. UT = Untreated.

We also monitored cucumin-induced apoptosis in the HPV-harbouring oral cell line by the expression of pro-apoptotic protein Bax and anti-apoptotic proteins (Bcl-2 and cIAP) (Figure 3). Interestingly, we observed downregulated anti-apoptotic markers (Bcl-2 and cIAP) with paralleled upregulated apoptotic protein (Bax) by curcumin suggesting the role of curcumin in apoptotis.

### Curcumin inhibits E6-mediated p53 degradation in HPV16-positive oral cancer cells

**Figure 4.**
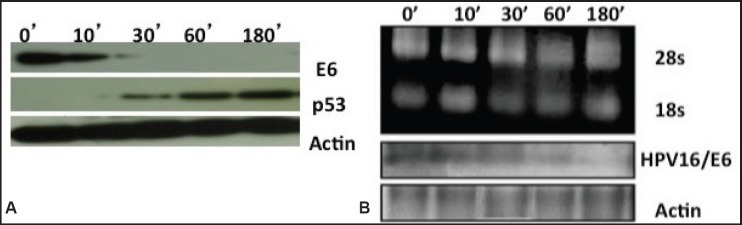
A) Immunoblotting showing reverse expression dynamics of HPV oncoprotein E6 and host p53 proteins in curcumin-treated cancer cells. Oncoprotein E6 decreased but host p53 increased with the increasing concentration of curcumin. GAPDH was utilised as loading control for protein samples. B) Northern blotting of HPV16/E6 and β-actin mRNA to detect their mRNA transcript levels. Decreased expression of E6 mRNAs seen post-curcumin treatment. Re-probing filters with β-actin cDNA probes confirmed equal loading of mRNA in each lane.

As the HPV16 viral E6 onoprotein is known to degrade the p53 host tumour suppressor during carcinogenesis, next we examined the role of curcumin on p53 expression by the HPV16-encoded E6 oncoprotein by western blotting (Figure 4A). As anticipated, we found that abolishment of the E6 oncoprotein expression resulted in the rescue of the p53 re-expression. Consistent with our observations of the northern blot (Figure 4B), the decline in E6 protein expression started at 30 minutes and completely disappeared by third hour. Again we have not found any modulation in the expression pattern of actin, confirming that the transcriptional suppression is not a general cellular event but specific to viral oncogenes. This result has indicated that curcumin treatment rescued p53 re-expression by negatively regulating E6 oncoprotein expression.

### Curcumin suppresses transcripton of oncogene E6 in HPV16-positive oral cancer cells

To find out the role of curcumin-mediated modulation on HPV transcription by altered DNA-binding pattern of host transcription factor complexes, we examined mRNA level of E6 viral oncogene.

Time- and concentration-dependent curcumin treatment studies revealed here that there was repression of the oncogene E6 mRNA transcription (Figure 4B). HPV16/E6 mRNA downregulation started after 30 minutes of 100 uM curcumin incubation in 93VU147T cells and completely disappeared by the third hour. This curcumin mediated abolition of HPV/E6 mRNA was indeed selective and virus-specific because endogenous host mRNA expression of actin gene was not suppressed.

Since the half-life of HPV16 mRNA is approximately 2.5 h as detected earlier in our laboratory [16], we have specifically restricted the analysis of the HPV-16/E6 transcripts to ≤3 h.

### Curcumin inhibits constitutive activation of AP-1, NF-kB but not Oct-1

**Figure 5.**
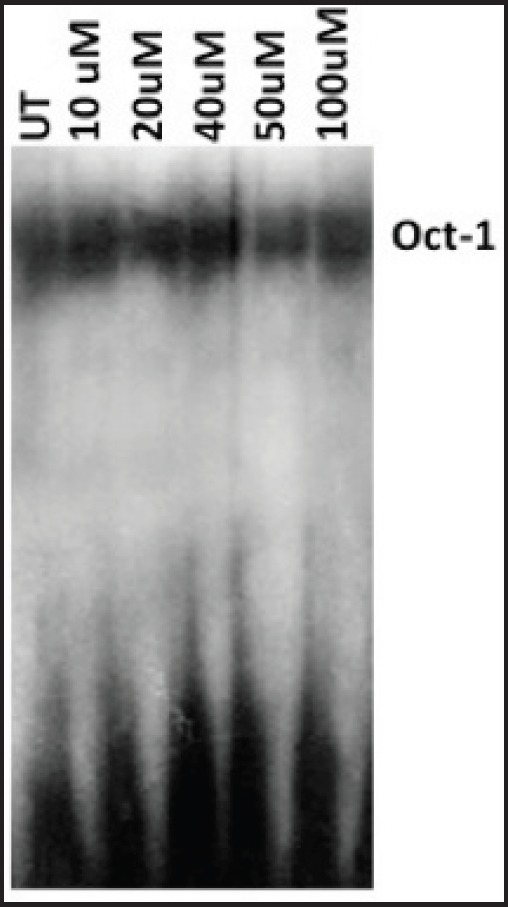
EMSA with radiolabelled [γ-^32^P] ATP. Oct-1 probe to show equal loading of nuclear extracts in cells treated with 0 to 100 uM curcumin. It also exhibits that curcumin does not effect transactivation of Oct-1, a constituent of basal transcriptional machinery. UT = Untreated.

**Figure 6.**
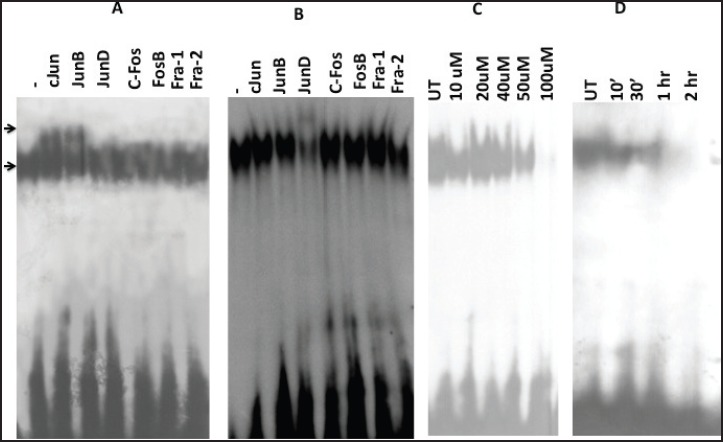
Alteration in the composition of DNA-binding AP-1 complex A. untreated cells B. curcumin-treated cells. Supershift analysis using nuclear extracts (10 ug) from oral cancer cells incubated with specific antibodies (2 ug each lane) either against AP-1 members C. Dose kinetics of curcumin in different concentrations (0–100 uM) on AP-1 trans-activation showing modified DNA binding D. Time kinetics of curcumin on AP-1 trans-activation at different time intervals (0–2 hr). UT = Untreated.

**Figure 7.**
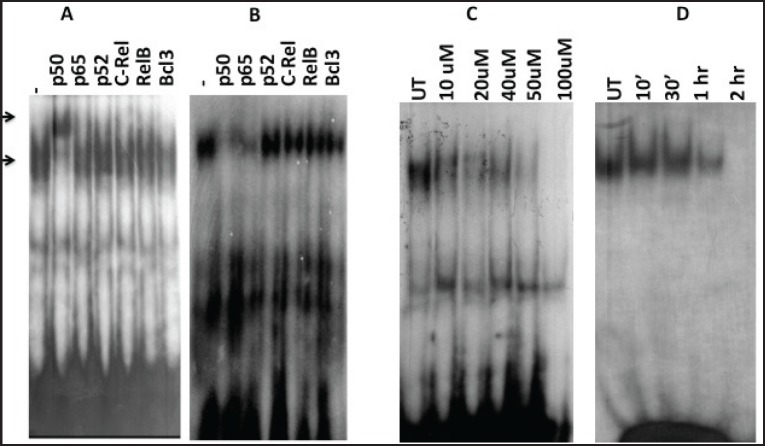
Alteration in the composition of DNA-binding NF-kB complex and reconstituted complex of NF-kB members in nuclear extracts in post-curcumin treatment. A) Curcumin untreated. B) treated cells. Supershift analysis using nuclear extracts (10 ug) from with specific antibodies (2 μg each) either against NF-kB members. C) Dose kinetics of curcumin on NF-kB activation. D) Time kinetics of curcumin on NF-kB activation. UT = Untreated.

Since the binding of redox-regulated transcription factors AP-1 and NF-kB in the cis-regulatory (URR) region of HPV modulates the transcription of downstream viral oncogene E6, we analysed the binding activities of these cellular factors in the nuclear extracts obtained from curcumin-treated cancer cells. The binding activity of basal transcription factor Oct-1 was not modulated by the treatment of curcumin (Figure 5). But the DNA-binding activities of AP-1 (Figure 6C) and NF-kB (Figure 7C) started declining after 50 uM and completely lost at 100 uM. Time kinetics studies showed that abolishment of transactivation of AP-1 (Figure 6D) and NF-kB (Figure 7D) started after 30 minutes of incubation with 100 uM curcumin by which time the HPV transcription was also found to be abolished. These data indicate that the specific inhibitory role of curcumin operates only on the activation of specific transcription factors and not on the basal transcription machinery of HPV.

### Curcumin modulates composition of DNA-binding complexes of AP-1 and NF-kB

To gain further insight into the modulation of HPV gene reguation by AP-1 and NF-kB host proteins in curcumin-treated state, we further dissected their composition by supershift assays. Interestingly, the composition of complex of both transcription factors was indeed changed after curcumin treatment of 100 uM for 1 h. Prior to curcumin incubation, AP-1 was composed of c-Jun/JunB (Figure 6A) but in post-treatment condition, it was changed to JunD/JunD homodimers (Figure 6B).

Similarly, the composition of NF-kB was observed to be changed from p50/p50 homodimer (Figure 7A) to p50/p65 heterodimeric (Figure 7B) DNA-binding complex in the presence of curcumin. These results together indicate the possible involvement of curcumin also on the alteration of composition of AP-1 and NF-kB complex during transcriptional regulation of HPV-16.

### Curcumin modulates the expression of AP-1 and NF-kB superfamily members in HPV-positive oral cancer cell line

**Figure 8.**
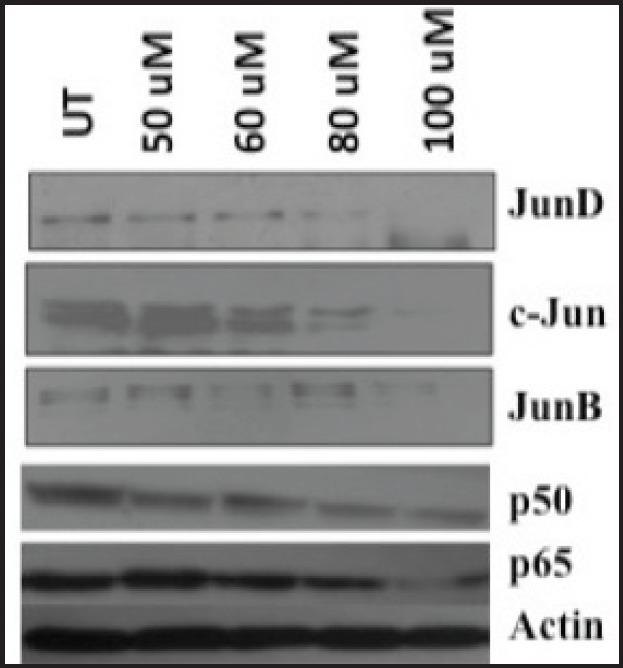
Immunoblotting showing differential expression pattern of proteins from AP-1 and NF-kB members in curcumin-treated cells (dose range = untreated to 100 uM). Decreased expression levels of c-Jun, JunD, JunB, p50, and p65 members after curcumin treatment. About 25–50 ug protein extracts of each treatment were resolved on an 8–20% gradient SDS-PAGE, electrotransferred on PVDF membrane and probed with different antibodies as described in method section. Equal total protein loading in each lane is confirmed by reprobing membrane with β-actin antibody. UT = Untreated. uM = micromolar.

Supershifting experiments have revealed that the c-Jun, JunB, and JunD members of the AP-1 super-family (Figures 6A and 6B) and the p50 as well as the p65 members of the NF-kB super-family (Figures 7A and 7B) were involved in the DNA-binding activity in pre- and post-curcumin treated cells. Therefore, we further analysed the expression pattern of only these specific members. Downregulation of the specific AP-1 and NF-kB proteins members’ expression started at 80 uM and was completely abolished at 100 uM (Figure 8). But the unaffected β-actin expressions level indicated that curcumin is specifically inhibiting the expression of AP-1 and NF-kB proteins in HPV-infected oral cancer cells (Figure 8). These findings suggest that the contribution of altered dimeric composition of transcription factors and their expression level in oral cancer cells are modulated by curcumin.

## Discussion

In the present study, the effect of curcumin on HPV16-induced viral oncogenesis in the oral cancer cell line is examined. Curcumin downregulates expression and activation of host transcription factors AP-1 and NF-kB bound at cis-regulatory region of HPV genome in a concentration- and time-dependent manner. This eventually suppresses HPV16/E6 transcription-mediated carcinogenesis.

Although the constitutive activation of AP-1 and NF-kB proteins is seen in various HNSCC cell lines [35, 36], the modulation of their transactivation and expression involved in HPV16-mediated OSCC with respect to any antioxidant is still an open area to study.

Two viral HPV—oncogenes E6 and E7—themselves possess intrinsic trans-activation capacity on their homologous promoters in transformed human keratinocytes but are mainly dependent on the availability of a defined set of host nuclear transcription factor on the cis-region of the HPV genome [2, 5]. Members of AP-1 and NF-kB transcription factors are required for efficient HPV16/18 transcription and considered central factors for transcription linked life cycle of HPVs [6–8].

A potential antioxidative agent curcumin (diferulolylmethane), an active component of the perennial herb turmeric (*Curcuma longa*), is extensively used in curry in the South East Asian region. It exhibits anti-viral and anti-tumour activity via multiple pathways of NF-kB and AP-1 [23]. It is a strong antioxidant and protects cells against free radical oxygen, that is, reactive oxygen species (ROS) damage. Retardation of tumorigenesis and reduction in DNA adducts by curcumin in oral cancer cells and leukoplakias have been also reported [19–21].

The present study demonstrates that curcumin treatment inhibits activation of NF-kB as well as AP-1 in HPV-positive cell by EMSA experiments. These results are in agreement with many previous reports [16, 29]. Interestingly, we noticed the change in the DNA-binding complex composition of transcription factors, which are well known to be reconstituted on DNA by redox regulation. In fact, DNA binding of NF-kB and AP-1 complex is altered by redox regulation by several mechanisms [12–14]. Based on our EMSA studies, we assumed that herbal antioxidant curcumin’s treatment had changed NF-kB composition from p50/p50 to p50/p65, although the participation of p65 was very minimal (Figure 7B). This might also indicate dimished p65-DNA-binding activity instead of shift in diner composition. However, more data are needed to further validate and explain this interesting altered recruitment pattern. The known repressive effect of p50/p65 heterodimer on HPV-16 transcription [8] explains here that how curcumin mediated change in NF-kB complex composition to p50/p65 hetrodimer negatively regulates HPV-16/E6 mRNA expression. Similarly, we also assume that post-treatment modifications of AP-1 complex from c-JunD/JunD to less active DNA binding homodimer JunD/JunD [27] may drive the HPV transcription machinery is less stronger manner leading to downregulated E6 mRNA expression.

The western blot data have also shown that curcumin downregulates the protein expression of AP-1 members: c-Jun, JunD, and JunB along with NF-kB members, p50 and p65, but not of endogenous actin. The protein expression profile and DNA-binding assays together indicate that curcumin regulates not only the expression level but also the DNA binding together with composition of the host cellular proteins in HPV16-infected oral cell line. Therefore, we anticipate that curcumin-mediated modulation in DNA binding of these two transcription factors is a consequence of altered composition as well as expression of their family members.

In this study, we have also shown that curcumin downregulates the expression of HPV16 E6 oncoprotein but simultaneously upregulates p53 tumour suppressor protein. This inverse correlation is completely consistent with earlier findings which had suggested that HPV-infected OSCC biopsies have wild type p53 isoforms [28, 31]. The suppression of E6 oncogene by an antisense approach and curcumin in HPV16-positive cervical cells Caski and in 47T oral cells have shown to restore apoptosis as well as re-appearance of p53 [21, 30, 31, 35].

Our northern blotting analysis has shown curcumin-mediated suppression of E6 mRNA transcripts. The band can be actually assumed to be a major transcript of ~ 1.5 Kb as reported earlier by Steenbergen *et al* [24]. The absence of other E6 viral transcripts of 2 and 4 kb are possibly due to their relative lower abundance. Half-life of HPV16 of ~ 3hrs determined in our laboratory earlier by the repression of HPV transcripts paralleled to the actinomycin-inhibited transcription [16]. These data together confirmed curcumin’s action on the transcriptional machinery in 93VU147T cells.

Curcumin-induced apoptosis in different epithelial cancer cells is shown earlier [23, 29, 32, 33] so we have examined the same in our HPV-positive oral cancer lines by utilising panels of some apoptotic and anti-apoptotic markers. We observed that curcumin is indeed capable of inducing apoptosis in HPV16-infected oral cancer cells-93VU147T. Reduced cIAP2 level may be explained by diminished recruitment of NF-kB and AP-1 on the promoter of cIAP2 gene [34]. But downregulation of Bcl-2 by curcumin might be due to altered NF-kB complex resulting in p650/p65 heterodimers on URR of HPV [8]. Therefore, in the light of the above data, we conclude that curcumin has therapeutic potential for suppression of HPV16-mediated oral oncogenesis.

## Conclusions

The herbal antioxidant curcumin has therapeutic potential for high-risk HPV-infected oral cancers. It downregulates HPV transcription via cellular transcription factors AP-1 and NF-kB in HPV-16-infected oral cancer cells.

## Conflicts of Interest

There is no conflict of interest.

## Authors’ contributions

Alok Mishra, Rakesh Kiumar, Abhishek Tyagi, Indu Kohaar, Suresh Hedau, Subhodeep Sarker, and Dipanker Dey performed experiments. Alok Mishra, Bhudev Das, Daman Saluja, and Alok C Bharti conceived the idea, wrote, and edited the manuscript.

## Abbreviations

HPV: Human Papillomavirus
AP-1: Activator Protein-1
NF-kB: Nuclear Factor Kappa B
